# Supplementary material for: Purifying selection against spurious splicing signals contributes to the base composition evolution of the polypyrimidine tract
Source: J Evol Biol. 2023 Aug 11;36(9):1295–312. doi: 10.1111/jeb.14205 (PMC10946897; doi:10.1111/jeb.14205)
Supplement: Supplementary file 1 — Appendix S1. [file JEB-36-1295-s001.pdf]

Supplement

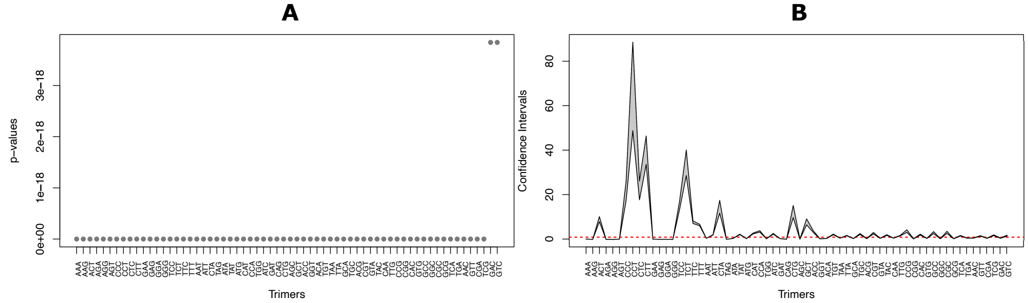

Figure S1: Tests of strand symmetric evolution in the 3PT. (A) Ordered p-values from chi-square tests, for the equality of forward and reverse complement trimers. (B) Confidence intervals for the ratios of forward and reverse complement trimers. Red dashed lines correspond to the tolerance range to assume equivalence.

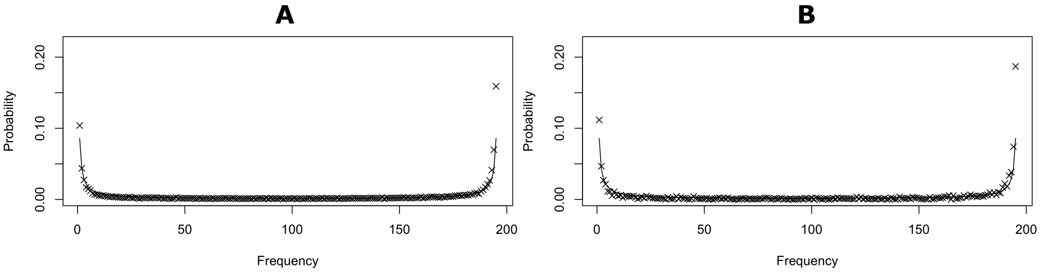

Figure S2: Biallelic spectra of GC-changing mutations (A/C, A/G, T/C, T/G) constructed based on their segregating GC frequency for (A) Autosomal short introns (B) X-linked short introns. For this analysis, only introns shorter than 66 bp were used. The lines represent expected site frequencies under neutral equilibrium; crosses represent observed site frequencies. The deviations are significant ($\chi^2_{df=194} = 1875.3$, $p < 0.001$ and $\chi^2_{df=194} = 518.81$, $p < 0.001$ for Autosome and X, respectively)

Table S1: Proportion of nucleotides (A, T, G, C) and mononucleotide asymmetry scores (S_{CG}, S_{TA}) in 5LR and 3PT of X-linked introns

	A	T	G	C	S_{CG}	S_{TA}
5LR	29.27%	31.45%	18.39%	20.89%	6.37%	3.58%
3PT	19.89%	44.70%	9.58%	25.83%	45.87%	38.41%

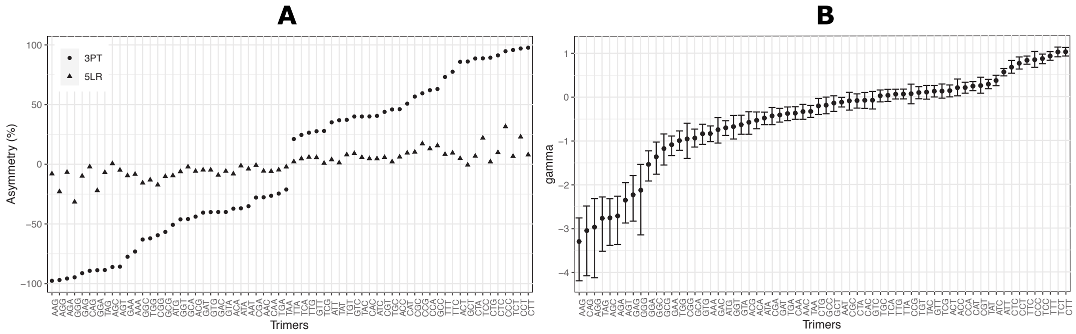

Figure S3: Results for X-linked introns. (A) Asymmetry scores per trimer, per region. Circles represent 3PT and triangles represent 5LR (B) Selection coefficients of each trimer in 3PT. Error bars represent the 95% CIs from 1000 bootstraps of the datasets.

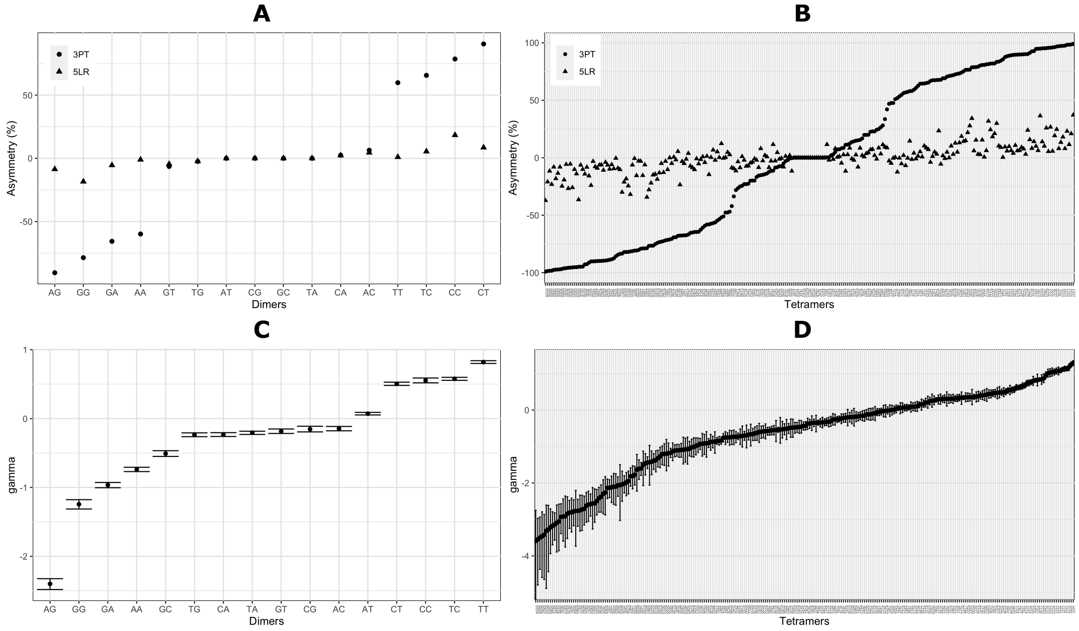

Figure S4: Results for dimers (A-C) and tetramers (B-D). (A-B) Asymmetry scores per region, per dimer and per tetramer, respectively. Circles represent 3PT and triangles represent 5LR (C-D) Scaled selection coefficients of each dimer and tetramer in the 3PT, respectively. Error bars represent the 95% CIs from 1000 bootstraps of the original datasets.

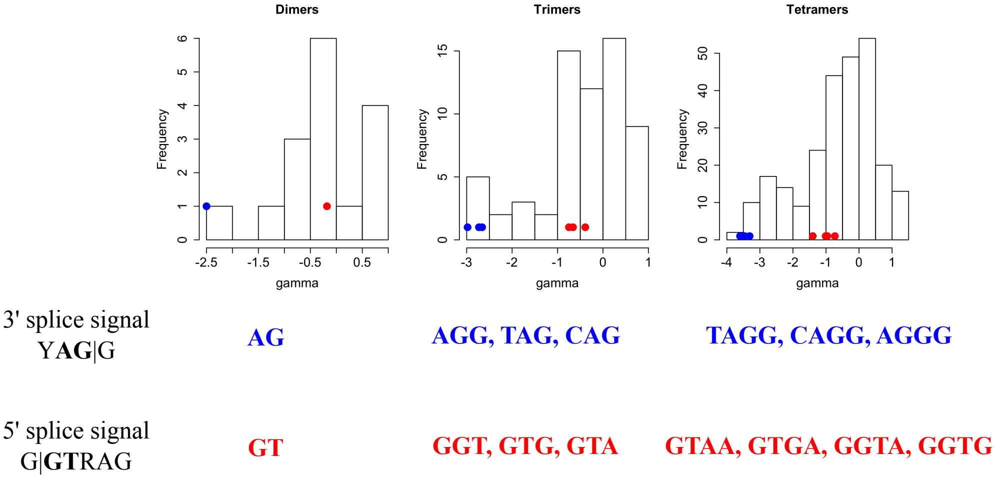

Figure S5: Scaled selection strength ($\gamma(M)$) distributions for dimer (16), trimer (64) and tetramer (256) motifs. Dots in the graphs correspond to the gamma values for specific motifs from 3' splice signal (blue) or 5' splice signal (red). The lower panel shows the pinned motifs in histograms.

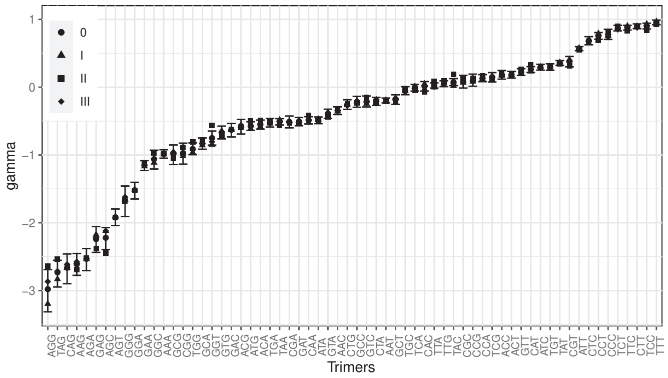

Figure S6: Scaled selection coefficients of each trimer ($\gamma(M)$) in the 3PT for different datasets. 0 (circle): original dataset with all short introns. I (triangle): dataset including only non-phase 0 (3n+1, 3n+2) introns. II (square): dataset including only phase 0 (3n) introns. III (diamonds): dataset excluding the common phase 0 introns between *D. melanogaster* and *D. simulans*. Error bars represent the 95% CIs from 1000 bootstraps of the original datasets.

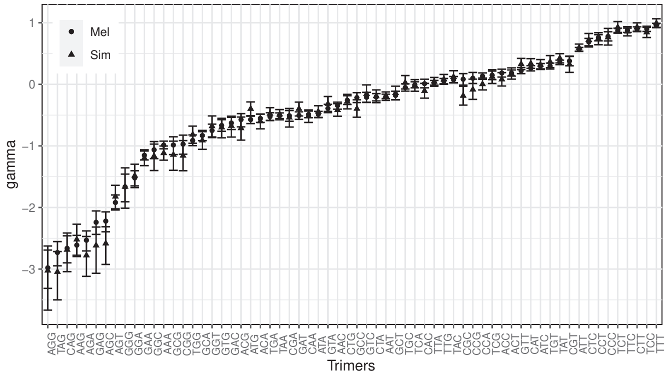

Figure S7: Scaled selection coefficients of each trimer ($\gamma(M)$) in the 3PT for different species. Circles are estimates from *D. melanogaster*, while triangles are estimates from *D. simuans*. Error bars represent the 95% CIs from 1000 bootstraps of the datasets.

Table S2: Likelihood ratio test statistic (LRT= -2*ln(likelihood ratio)) for different models fit to autosomal data. Differences in number of parameters are given as the degrees of freedom in brackets. Values are significant for all positions and comparisons. Both HI and HIII fit significantly better than HII, and HIII fits significantly better than HII.

Position	HI vs HII (df=2)	HIII vs HI (df=1)	HIII vs HII (df=3)
1	142.28	283.29	425.58
2	340.16	254.37	594.54
3	624.80	290.38	915.18
4	662.60	377.02	1039.62
5	426.75	440.11	866.86
6	376.55	457.91	834.46
7	1567.81	317.51	1885.32
8	2838.01	290.37	3128.37
9	4457.30	90.78	4548.07

Table S3: Likelihood ratio test statistic (LRT= -2*ln(likelihood ratio)) for different models fit to X chromosomal data. Differences in number of parameters are given as the degrees of freedom in brackets. Values are significant for all positions and comparisons. Both HI and HIII fit significantly better than HII, and HIII fits significantly better than HII.

Position	HI vs HII (df=2)	HIII vs HI (df=1)	HIII vs HII (df=3)
1	43.55	52.84	96.39
2	34.06	46.09	80.15
3	55.98	67.98	123.95
4	82.40	60.82	143.22
5	65.87	67.41	133.28
6	39.52	73.20	112.72
7	228.29	44.89	273.18
8	260.64	44.90	305.55
9	525.97	21.12	547.10

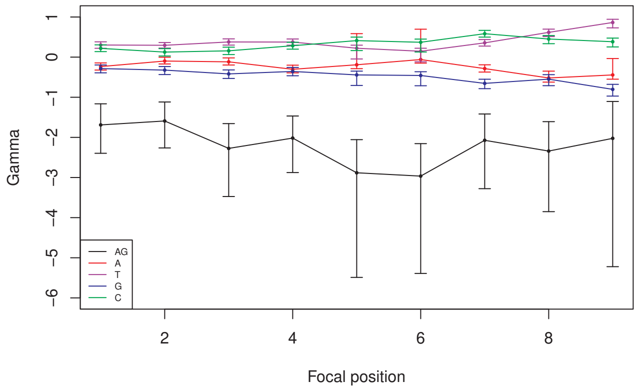

Figure S8: Inferred scaled selection coefficients γ of the monomers and dimer AG for each position in the X-linked 3PT under the model HIII. Error bars represent 95% CIs from 1000 bootstraps of the datasets.

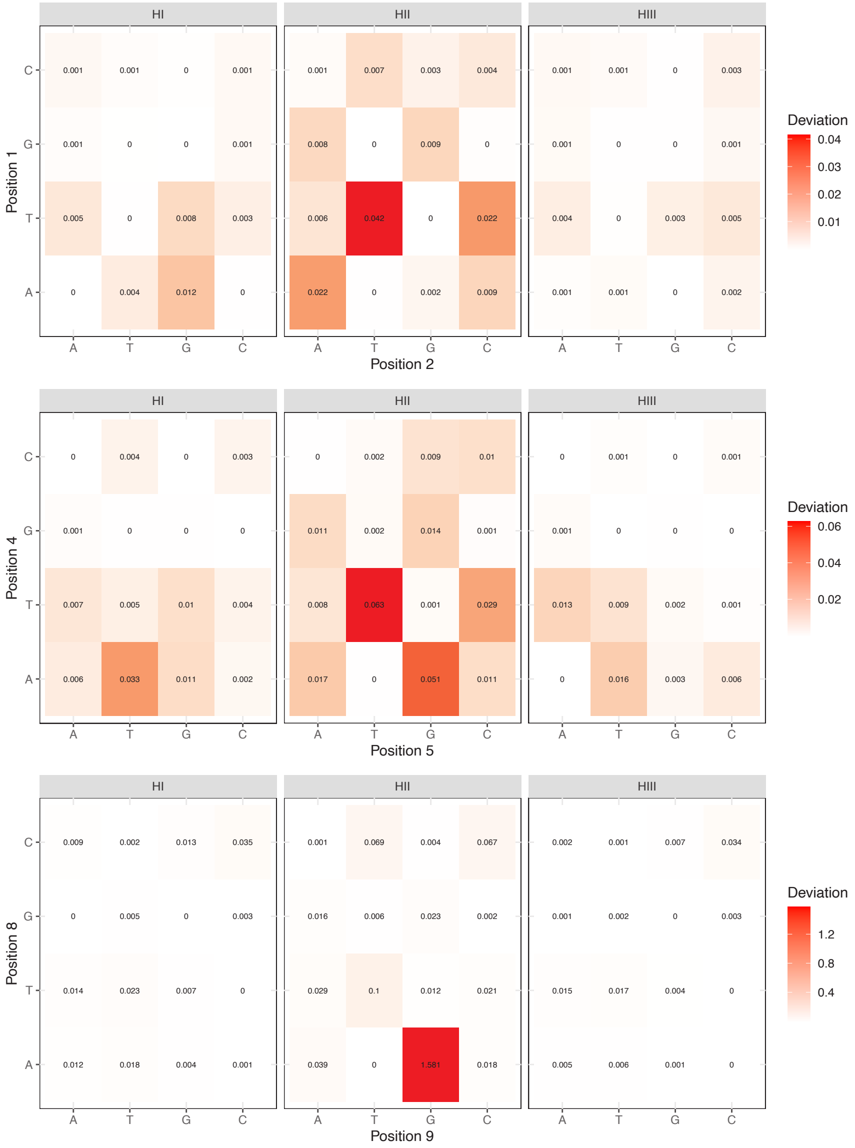

Figure S9: Deviations of the three hypothesis from the X chromosomal empirical joint frequency data of the four bases. Values calculated as χ^2 statistic and high to low deviation is represented with red to white colour gradient. Matrices for three positions are chosen to visualize the pattern along the 3PT .

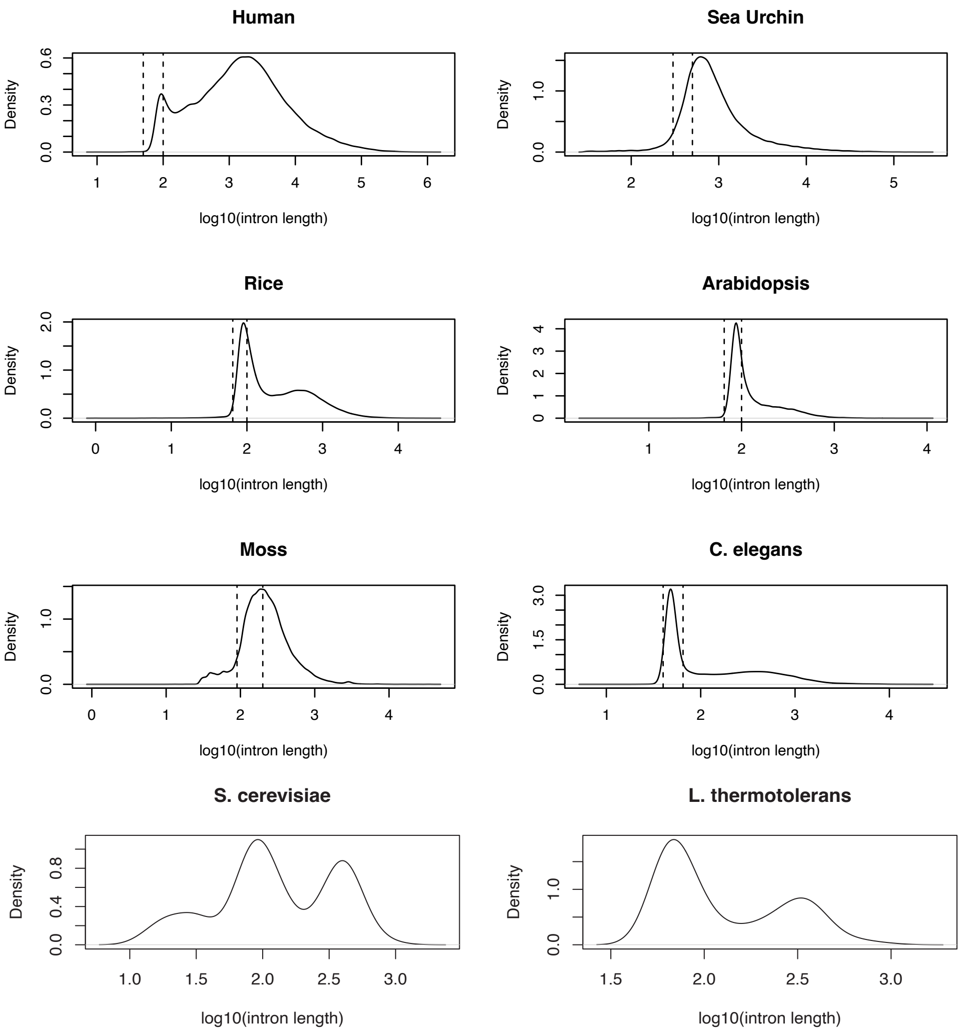

Figure S10: The length distribution of introns obtained for eight eukaryotic species. Vertical dashed lines represent the length range of extracted and analyzed short intron class. Human: 50-100 bp, Sea Urchin: 300-500 bp, Rice: 65-100 bp, *Arabidopsis*: 65-100bp, Moss: 90-200 bp, *C. elegans*: 40-65 bp. For yeast species all annotated introns were utilized.

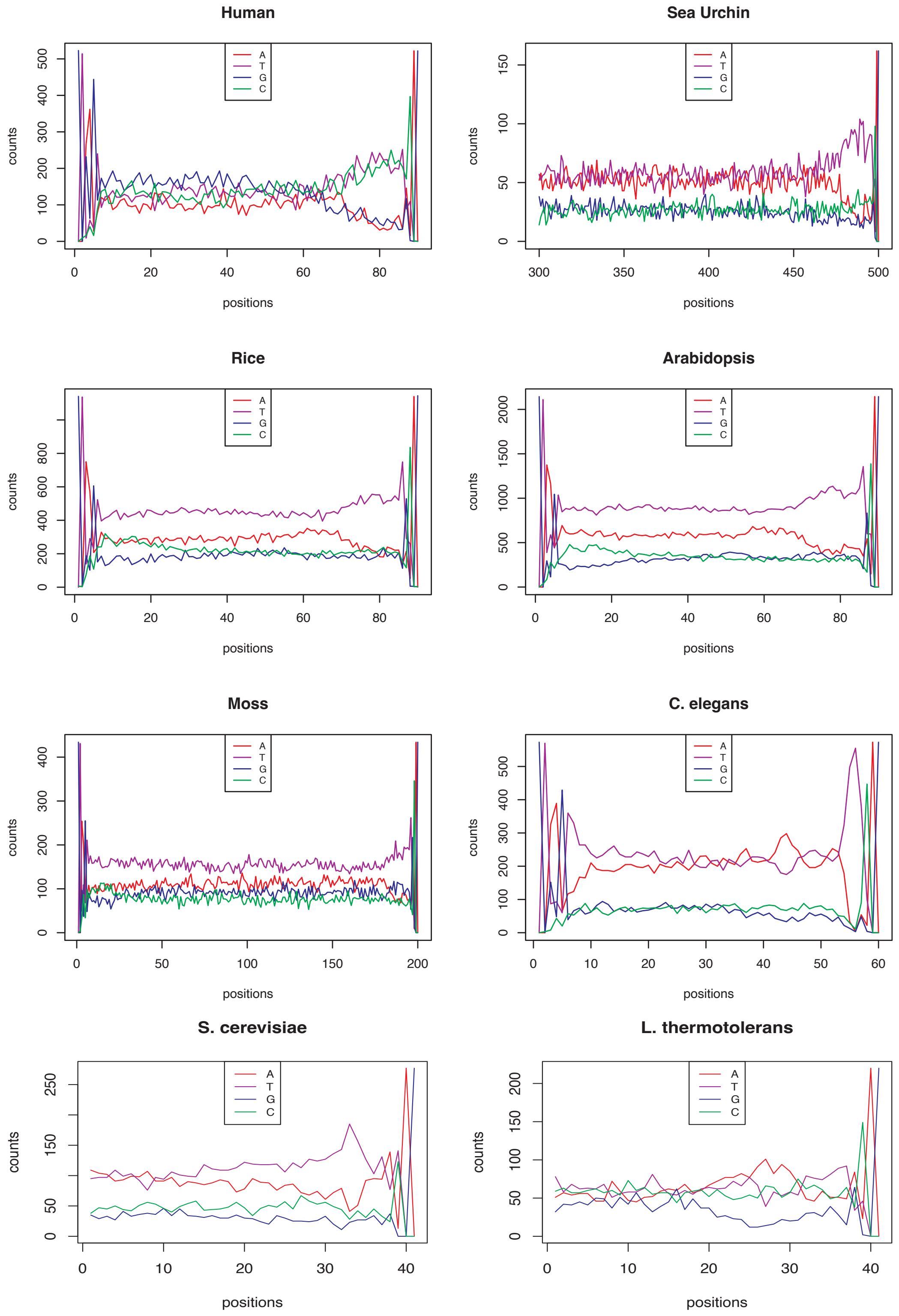

Figure S11: Nucleotide composition of introns with length 90 for human, 500 for sea urchin, 90 for rice and *Arabidopsis*, 200 for moss, and 60 for *C. elegans*. Only one candidate length class were chosen to visualize pattern. Total numbers of introns used for each species are 523, 162, 1152, 2145, 434 and 573, respectively. For yeast species all annotated introns with different lengths were utilized (277 and 220 introns for *S. cerevisiae* and *L. thermotolerans*, respectively), thus nucleotide composition is given for the last 40 bases from the 3' end.

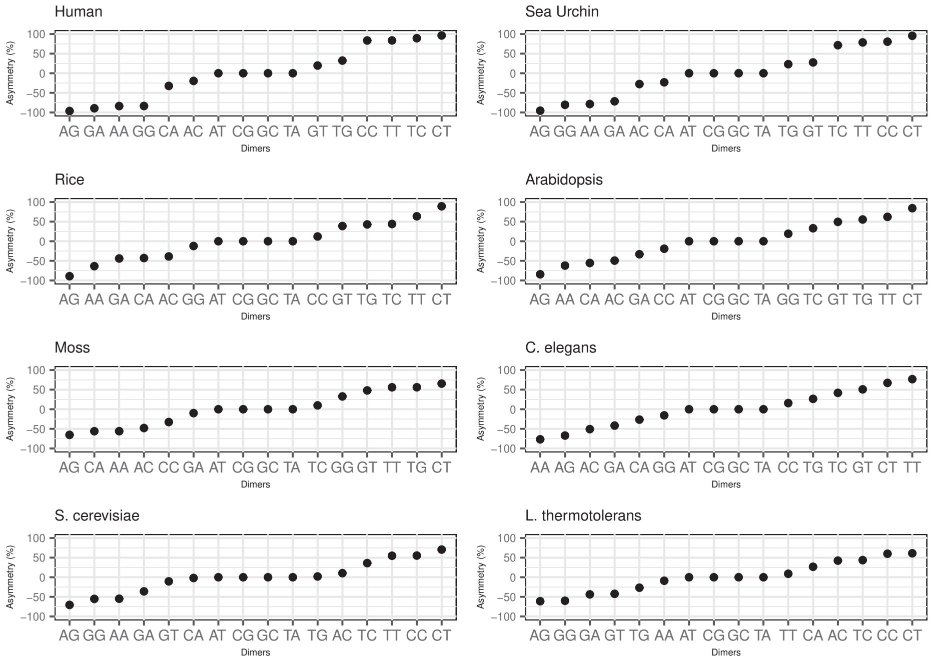

Figure S12: Asymmetry scores of dimers from the pyrimidine enriched regions in the introns of 8 eukaryotic species.

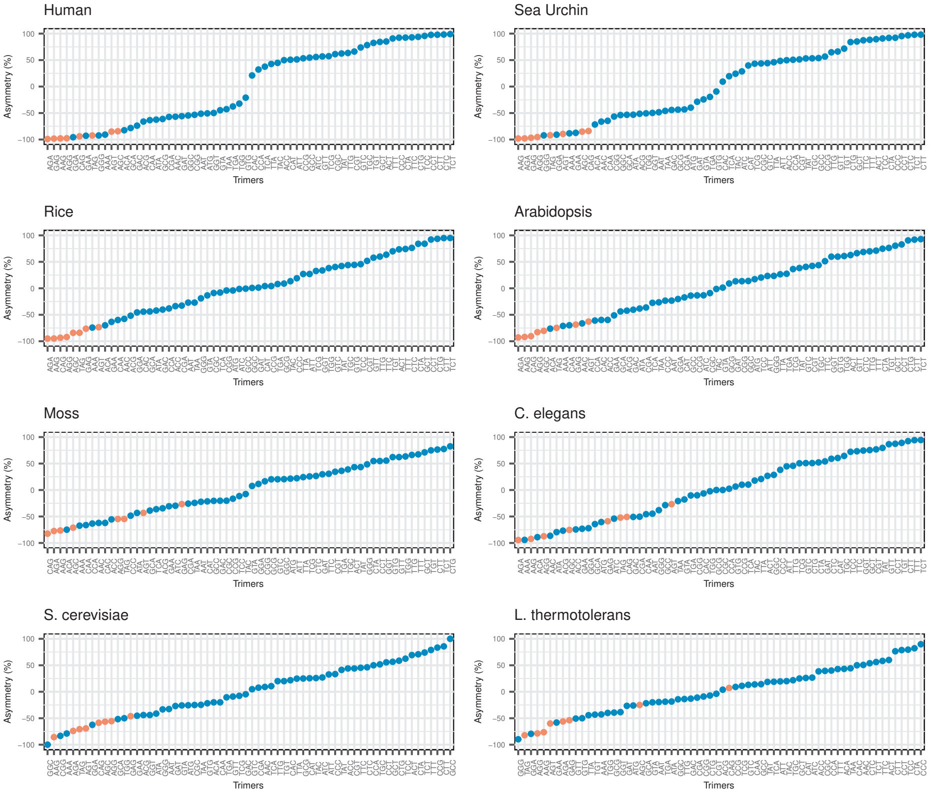

Figure S13: Asymmetry scores of trimers from the pyrimidine enriched regions in the short introns of 8 eukaryotic species. Motifs containing AG in it are depicted with red points, while non-AG motifs are represented with blue.
